# Identification and Characterization of a Novel Emaravirus Associated With Jujube (*Ziziphus jujuba* Mill.) Yellow Mottle Disease

**DOI:** 10.3389/fmicb.2019.01417

**Published:** 2019-06-25

**Authors:** Caixia Yang, Song Zhang, Tong Han, Jingjing Fu, Francesco Di Serio, Mengji Cao

**Affiliations:** ^1^Liaoning Key Laboratory of Urban Integrated Pest Management and Ecological Security, College of Life Science and Engineering, Shenyang University, Shenyang, China; ^2^National Citrus Engineering and Technology Research Center, Citrus Research Institute, Southwest University, Chongqing, China; ^3^Academy of Agricultural Sciences, Southwest University, Chongqing, China; ^4^Istituto per la Protezione Sostenibile delle Piante, Consiglio Nazionale delle Ricerche, Bari, Italy

**Keywords:** jujube (*Ziziphus jujuba* Mill.), high-throughput sequencing, *Emaravirus*, transmission electron microscopy, phylogenetic relationship, field survey

## Abstract

A previously unreported disease affecting jujube (*Ziziphus jujuba* Mill.) trees was observed in China (Liaoning province) in 2015 and named jujube yellow mottle disease (JYMD), due to prevalent symptoms on the leaves. Diseased plants produced also malformed and discolored fruits. In an attempt to identify the possible causal agent of JYMD, high-throughput sequencing of small RNA libraries was performed and a novel virus, tentatively named jujube yellow mottle-associated virus (JYMaV), was identified and characterized. Six genomic RNA segments of JYMaV were completely sequenced. Each one contains a single open reading frame in the viral complementary strand and two untranslated regions with complementary 5′ and 3′ terminal ends, thus showing typical features of other negative-stranded RNA viruses. RNA1 (7.1 kb), RNA2 (2.2 kb) and RNA3 (1.2 kb) encode putative proteins that, based on their conserved motifs, have been identified as the RNA dependent RNA polymerase, the glycoprotein and the nucleocapsid protein, respectively. These proteins share significant sequence identity (52.1–70.4%) with proteins encoded by raspberry leaf blotch virus (RLBV). RNA4 (1.5 kb) and RNA5 (1.2 kb) code for two putative 30 K movement proteins also related to the homologous RLBV protein. The functional role of the protein encoded by JYMaV RNA6 remains unknown. These data together with the phylogenetic relationships of JYMaV with other recognized emaraviruses support the proposal that JYMaV is the representative member of a novel species in the genus *Emaravirus*. In agreement with this proposal, virus-like particles and double-membrane-bound bodies, similar to those previously reported for other emaraviruses, were observed by transmission electron microscopy in extracts and tissues from symptomatic leaves, respectively. A specific RT-PCR-based detection method has been developed and used in a preliminary field survey that provided results strongly supporting the close association of JYMaV with the novel disease.

## Introduction

European mountain ash ringspot-associated virus (EMARaV), rose rosette virus (RRV), fig mosaic virus (FMV), pigeonpea sterility mosaic virus and pigeonpea sterility mosaic virus-2 (PPSMV and PPSMV-2), High Plains wheat mosaic virus (HPWMoV), raspberry leaf blotch virus (RLBV), redbud yellow ringspot-associated emaravirus (RYRaV), and actinidia chlorisis ringspot-associated virus (AcCRaV)^1^ (Mielke-Ehret and Mühlbach, 2007; Elbeaino et al., 2009a,b, 2014, 2015; Laney et al., 2011; Ishikawa et al., 2012; McGavin et al., 2012; Tatineni et al., 2014; Di Bello et al., 2015, 2016; Lu et al., 2015; Zheng et al., 2016) are representative members of the nine virus species included in the genus *Emaravirus* of the family *Fimoviridae*, order *Bunyavirales* (Maes et al., 2019). The recently discovered blackberry leaf mottle-associated virus (BLMaV) (Hassan et al., 2017) and Palo verde witches broom virus (PVWBV) (Ilyas et al., 2018) have *Emaravirus* attributes but they have not been officially classified yet. These plant-infecting viruses have various modes of transmission including eriophyid mites, seeds (rare), grafting, mechanical friction and contaminated cutting implements. In most cases, spherical virions enveloped by a double-membrane are observed in the infected plant cells (Mielke-Ehret and Mühlbach, 2012; Patil and Kumar, 2015). Although not all emaraviruses have been biologically and morphologically characterized, they typically have a genome composed of multiple (four to eight) negative-sense RNAs, encoding at least an RNA-dependent RNA polymerase (RdRp), a glycoprotein (GP) precursor, a nucleocapsid protein (NP) and a nonstructural movement protein (MP) as core elements (Elbeaino et al., 2018). The functional role of the proteins encoded by other RNAs remains generally unknown, with a suggested possible involvement in viral pathogenicity and suppression of plant antiviral defense based on RNA silencing (Lu et al., 2015; Gupta et al., 2018). In the case of RLBV, it has been shown that these non-structural proteins may interact with each other and/or with the NP, with evidence of their involvement in viral pathogenesis provided for some of them (Lu et al., 2015). Yet, it is unclear how many RNA components are necessary for the emaravirus infectivity.

The jujube (*Ziziphus jujube* Mill., family Rhamnaceae) grows mostly in Europe, Australia, and China (Gao et al., 2013). This fruit tree species is widely planted in most temperate zones of China. Excluding a disease associated with a jujube mosaic-associated virus (JuMaV), a virus recently identified by Du et al. (2017), the virus diseases in jujube have not been extensively studied. In 2015, jujube trees with leaves and fruits showing severe symptoms of a disorder never observed previously and named jujube yellow mottle disease (JYMD), were found in Chaoyang City (Liaoning province, China). In this study, a new negative-stranded RNA virus, identified in JYMD-affected jujube plants by high-throughput sequencing (HTS), is reported. Sequencing of the full length genome, observation of virus particles by transmission electron microscopy (TEM) and phylogenetic analyses support the proposal that such a virus is the representative member of a new species in the genus *Emaravirus*. The tentative name jujube yellow mottle-associated virus (JYMaV) is proposed for this virus because a preliminary field survey provided solid evidence of its close association with JYMD.

## Materials and Methods

### Plant Materials

**FIGURE 1:**
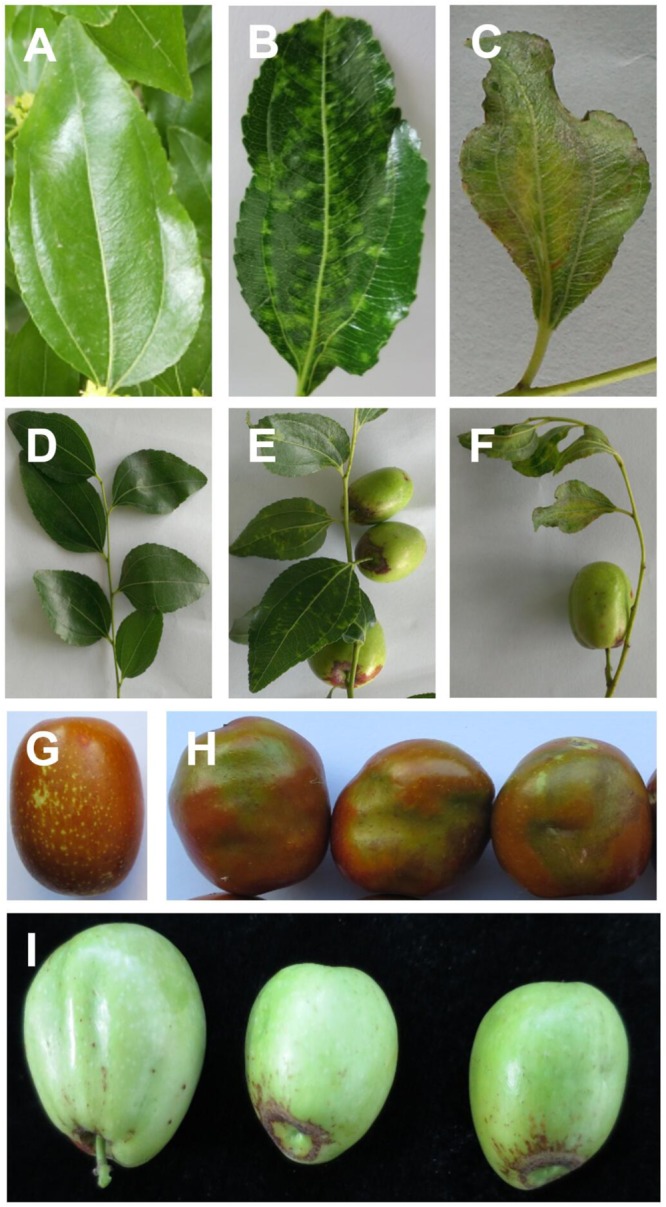
Symptoms of jujube infected with jujube yellow mottled-associated virus (JYMaV), on leaves **(B,C)**, fruit spurs **(E,F)**, mature and young fruits **(H,I)**, comparing with healthy leaf **(A)**, fruit spur **(D)**, and mature fruit **(G)**.

Leaf samples were collected from one symptomatic jujube tree grown in an orchard in Chaoyang County, Chaoyang City, Liaoning province, China, stored at -80°C and used for small RNA and transcriptome HTS (sRNA-seq and RNA-seq, respectively). Leaf samples from an asymptomatic tree (Figure 1A,D,G) were used as negative controls in the sequencing analyses. The same samples were also examined by TEM. Young fruits and leaves from symptomatic or asymptomatic trees were used as source material during the survey.

### Small RNA Sequencing

Total RNA (TNA) from symptomatic and asymptomatic leaves was extracted using a hexadecyltrimethylammonium bromide (CTAB)-based method. A TNA aliquot (3 μg) was separated by PAGE (Cao et al., 2014) and, using the 14–30 ssRNA Ladder Marker (TAKARA, Japan) as molecular marker, small RNAs with a size between 14 and 30 nt were recovered from the gel. The sRNA library was prepared with a NEBNext^®^Multiplex Small RNA Library Prep Set (New England BioLabs^®^Inc., United States) and sequenced using the BGISEQ-500RS (Huada Gene, Shenzhen, China). Raw reads data for the symptomatic and asymptomatic samples were processed to trim the adaptors and low-quality reads (quality score limit = 0.05; maximum number of ambiguities = 2; reads below 18 and above 30 nt discarded) were filtered out (Zhang et al., 2018). High quality reads were further filtered to remove host reads by mapping them to the *Ziziphus jujuba* (common jujube) genome^2^ (Liu et al., 2014). Retained reads were assembled *de novo* into larger overlapping sequences (contigs) using Velvet software 0.7.3 (Zerbino, 2010). Output sRNA contigs were used to search for similar sequences available in NCBI databases using BLAST (x and n) analysis^3^.

### Reconstruction of the Viral Genome

A preliminary scaffold of the novel virus was generated by aligning the viral contigs identified in the sRNA library from jujube with the corresponding RLBV genomic segments. The sequence gaps between the aligned contigs were filled by RT-PCR using virus-specific primers. The primers design strategy, and the position of the expected amplicons in the complete viral genomic RNAs are shown in Supplementary Figure S1. The 5′- and 3′-end sequences of viral genomic RNAs were determined by the rapid amplification of cDNA ends (RACE) technique using a GeneRacer kit (Invitrogen, United States) and gene specific primers (GSP) designed according to manufacturer’s recommendations (Supplementary Table S1). The generic terminal primer set Em-5F (GGATCCAGTAGTGTTCTCC)/Em-3R (GACTCG AGTAGTGAACTCC) has been used to directly amplify genomic RNA segments of JYMaV and to check for the existence of additional viral genomic components (Zheng et al., 2016). For sequencing purposes, PCR amplified products were purified from agarose gels using a TIANgel Midi Purification Kit (Tiangen, Beijing, China) and ligated into the pMD18T vector (TaKaRa, Dalian, China), which was used to transform competent *E. coli* DH5α cells. Five clones per amplicon were sequenced in both directions by the BGI Company (Beijing, China).

### Verification of Viral Genomes by Transcriptome Sequencing

Total RNA was extracted from leaves sampled from symptomatic and asymptomatic jujube trees using TRIzol reagent (Invitrogen, United States) according to manufacturer’s instructions. Purity, concentration and integrity of the extracted RNAs were tested by Nanodrop (Thermo Fisher Scientific, United States), Qubit 3.0 (Invitrogen, United States), and Agilent2100 (plant RNA Nano Chip, Agilent, United States). After rRNAs depletion, an RNA library was built using a TruSeq RNA Sample Prep Kit (Illumina, San Diego, CA, United States), sequenced by Illumina Hiseq (Mega genomics, Beijing, China) with pair-ended (PE) reads length layout 150 bp. Raw sequencing data were cleaned of low quality sequences by the CLC Genomics Workbench 9.5 (Qiagen, Valencia, CA, United States). Reads without sequence similarity, and not mapping to the reference jujube genome were assembled *de novo* by Trinity program (Grabherr et al., 2011). The generated contigs were used as queries for BLAST searches; contigs that were not identified as sequences already included in the databases were sorted out as candidate genomic fragments of the novel virus.

### Sequence Analysis

The ORF finder^4^ was used to predict open reading frames (ORFs) in both polarity strands of viral RNAs. The Conserved Domain Database (CDD)^5^, and ANNIE (Ooi et al., 2009)^6^ were used for a comprehensive annotation, such as conserved domains, coils, signal peptides, and transmembranes (TM), of putative viral proteins. PROMALS3D^7^ was used for protein secondary structure prediction (Pei et al., 2008). Pairwise percent nucleotide and amino acid identity between the novel virus and related viruses were computed by the CLC Genomics Workbench 9.5. Phylogenetic analysis of the RNA 1–4 coding regions was performed by MEGA 7.0 (Kumar et al., 2016).

### Transmission Electron Microscopy (TEM) Observations

Fresh young leaves of symptomatic and symptomless jujube trees previously used for the preparation of the RNA libraries sequenced by HTS, were individually sampled to perform TEM observations. Negative staining was used to observe the morphology and size of viral particles. The leaves were placed in phosphate buffer (PB, 0.1 mol/L, pH 8.0), homogenized and centrifuged at 9,000 r/min for 8 min. Copper grids with supernatant adsorbed (5 min), stained in 2% w/v phosphotungstic acid (pH 6.7; 10 s), were observed under TEM (Hitachi TEM system, Tokyo, Japan) and photographed. To study the distribution of virions in host cells and the degree and characteristics of cell lesions, ultrathin sections were used. Leaves with typical symptoms were cut into 4 mm × 2 mm slices and fixed in 5% w/v glutaraldehyde overnight. They were then rinsed twice with PB solution and fixed in 1% osmium tetroxide for 2 h, followed by ethanol gradient dehydration and embedding. Ultrathin sections were double-stained with 2% w/v lead citrate and 5% uranyl acetate, and examined with a TEM (Martelli and Russo, 1984).

### Virus Detection and Field Survey

For detection purposes, total nucleic acid was extracted from jujube leaves and/or fruits using the CTAB method. Two step RT-PCR with the primer set R3NP-R: GCAGCAATACAAATAGCCATG (nt position at 759–779)/R3NP-F: CACTCAACTTGGCAACAGTATC (nt position at 983–1004) targeting the NP gene was used for sensitive detection of JYMaV in jujube leaves and/or fruits. The primers were designed in a region with low sequence variability based upon sequencing data generated in this study. The field survey was performed by testing 45 and 15 symptomatic and symptomless jujube trees sampled from different planting areas of Liaoning province, respectively. Eighteen symptomless trees grown in Hebei province, where the disease was not observed so far, were also tested. To exclude the infection by a badnavirus, the jujube trees used for generating the HTS libraries were assayed by PCR using two generic degenerate primers (BadnaFP and BadnaRP) designed to amplify most known badnaviruses, as described previously (Yang et al., 2003).

Reverse transcription (RT) was conducted as previously described and conventional PCR was performed in a final volume of 25 μl, containing 10× buffer (2.5 μl), Ex Taq (Takara, 0.2 μl), F/R primers each (1 μl of 10 μM stock solution), dNTPs (1 μl of 10 mM stock solution), cDNA templates (2 μl) and dd H_2_O 17.3 μl. The PCR program consisted of 5 min initial denaturation at 95°C, followed by 35 cycles of 30 s denaturation, 30 s annealing at 55°C and 30 s extension at 72°C, and a final 7 min extension at 72°C.

## Results

### Symptom Description

In September 2015, jujube trees displaying symptoms of a disease apparently not reported previously were found in Chaoyang City, Liaoning Province. The prevalent symptoms were observed on leaves that showed mottling, yellowing and distortion (Figure 1B,C,E,F), and on fruits that were distorted, malformed, and discolored, with necrotic areas mainly around the calix (Figure 1H,I). According to the prevalent symptomatology on the leaves, the disease was named JYMD. Preliminary attempts to isolate and culture possible bacterium or fungus involved in the etiology of the disease were unsuccessful. Moreover, the previously described badnavirus JuMaV was not detected by PCR in symptomatic plants (data not shown). Therefore, the possibility that another virus or viroid was responsible for the disease was further investigated by next-generation sequencing approaches.

### Virus Identification

sRNA-seq was performed using RNA preparations from symptomatic and symptomless trees. A total of 21,067,489 clean reads (ranging from 18 to 30 nt) was generated from the small RNA library of symptomatic jujubes. The 21-nt reads were the most abundant, followed by those of 22- and 24-nt. Results of the BLAST searches of the 653 contigs (ranging in size from 50 to 372 nt) assembled from 4,411,092 non-host reads showed that 72 shared significant sequence similarity with RNAs of emaraviruses (e-values: 1.33E-47–8.76E-3); BLASTx searches restricted to the taxid *Emaravirus* increased the number of potential viral contigs to 99 (e-values: 3.53E-51–9.79E-03). No contig suggesting the presence of other viruses was found in the sRNA library from symptomatic jujube tree and no viruses were identified in the asymptomatic jujube. These data strongly suggested that the identified contigs in the symptomatic jujube trees could correspond to the genomic components of one (or more) novel emaravirus, provisionally named JYMaV. To confirm the presence of the viral sequences in the symptomatic jujube trees, TNA from symptomatic and non-symptomatic plants were further tested by RT-PCR using specific primers designed in several viral contigs (Supplementary Figure S1). Amplicons of expected size were only obtained from TNA extracted from symptomatic jujube trees (data not shown), thus confirming an ongoing infection by an emaravirus in these plants by an independent detection method.

### Primary Determination of the Virus Genome

**FIGURE 2:**
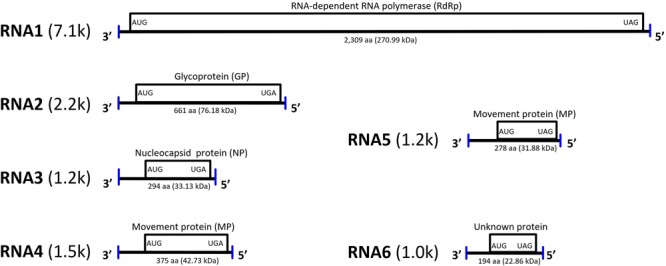
Genome organization and gene expression of jujube yellow mottled-associated virus (JYMaV). The proteins encoded by each RNA are represented as boxes. Vertical lines at the genomic ends indicate the conserved 13 nt at each terminus.

Blast searches revealed that the viral contigs identified in jujube trees had the highest nucleotide and amino acid sequence identity with genomic components of RLBV (65.6–78.8 and 34.4–71.0%, respectively) that, therefore, were used as references to generate preliminary scaffolds for the JYMaV genomic fragments (Supplementary Figure S1). Based on such scaffolds, specific JYMaV primers were designed (Supplementary Table S1) to amplify, clone and sequence overlapping and terminal cDNA fragments of JYMaV RNAs. The viral cDNAs were never amplified from symptomless jujubes. This strategy allowed the Sanger-based conventional sequencing of six JYMaV full-length RNAs (RNA1–6, Figure 2) that showed almost identical terminal ends, a feature typical of emaraviruses and other nsRNA viruses.

Therefore, as for other emaraviruses (Mielke-Ehret and Mühlbach, 2012; Zheng et al., 2016), primers designed in the conserved terminal sequences are expected to simultaneously amplify all or most viral genomic components. Interestingly, when primers with these features (Supplementary Table S1) were used with TNA preparation from jujube trees, simultaneous amplification of cDNAs with sizes consistent with those of five genomic components of JYMaV were generated in samples from symptomatic plants (Supplementary Figure S4). More specifically, conspicuous bands with sizes consistent with those expected for the full length cDNAs of JYMaV RNA 2–6 were observed in the agarose gel (Supplementary Figure S4). Cloning and sequencing of these amplicons confirmed that they actually corresponded to JYMaV RNAs from 2 to 6. cDNAs of RNA1 were not detected by this assay, possibly due to inefficient RT-PCR amplification of the large-sized genomic RNA template (7.1-kb). A non-expected cDNA of about 0.7 kb (Supplementary Figure S4) was also amplified; after sequencing, it resulted to be a non-specific amplicon originated from a host RNA (data not shown). No additional viral RNA was found by this analysis. This finding supports the view that JYMaV has a genome composed of only six segments. The same amplicons were consistently generated when TNAs extracted from at least twelve different symptomatic jujube trees were used in parallel analyses, thus confirming the close association of the six JYMaV genomic component with each other and providing evidence that they belong to the same viral entity. The JYMaV genomic RNA1 to RNA6 full-length sequences were submitted to NCBI database and assigned GenBank accession numbers from MK305894 to MK305899.

### Further Confirmations of the Viral Genome

Raspberry leaf blotch virus, the closest relative virus of JYMaV, has a genome composed of eight genomic RNAs. In contrast, assembling of sRNA reads only allowed the identification of six viral RNAs, five of which are closely related with RLBV. Therefore, at this stage, it could not be excluded that other JYMaV genomic components remained undetected. One possible reason could be that small RNAs deriving from possible additional genomic components accumulate at lower level with respect to those already identified, thus impairing the assembling of sRNAs into contigs of significant length. To further investigate the possible existence of additional genomic segments by a different HTS strategy, a rRNA-depleted library of total RNAs was generated from the same symptomatic jujube tree already tested by sRNA-seq and sequenced. RNA-seq produced a total of 73,659,744 clean reads, 70,677,145 of which (95.95%) were removed because mapping in the reference jujube genomes. Finally, 2,982,599 reads were assembled and 16,077 contigs (from 200 to 7,132 nt long) generated. After BLASTx analyses, all the contigs related to known RLBV genomes were excluded and the remaining contigs were further screened. However, this search did not allow us to find other potential plant viral genomic RNA components besides the reported six JYMaV RNAs.

### Viral Genome Characterization

The overall RNA molecules of JYMaV contain: (i) a single ORF in the viral complementary (vc) strand, (ii) 5′ and 3′ untranslated regions (UTRs) with respective lengths of 112–321 and 57–101 nt that share highly conserved nucleotides at their terminal 13 positions, and (iii) highly complementary nucleotide stretches (18–20) nt at both genomic termini that form a panhandle structure (Kormelink et al., 2011). These features are all consistent with those found in other members of the genus *Emaravirus* (Elbeaino et al., 2018).

RNA1 encodes a putative protein (P1) of 2,309 aa (ORF1; nt 7,042–113), with a predicted molecular weight of 270.99 kDa. P1 contains the RNA-dependent RNA polymerase domain of Bunya_RdRp super family (pfam04196, e-value: 5.91e-44) between aa 656 and 1436. Sequence analysis revealed five sequential signature motifs in the P1 with a typical arrangement [A (D_1137_ASKW), B (Q_1223_GNLNATSS), C (S_1264_DD), D (K_1308_K), E (E_1318_FLSS)] of the core RdRp regions of members of the genus *Emaravirus* (Supplementary Figure S2) (Laney et al., 2011; Elbeaino et al., 2015). In addition, P1 contains also an endonuclease domain in the N-terminus (H_118_D-P_157_D-EVK_174_), deemed to involve cap-snatching of viral mRNAs during genome expression (Juan et al., 2010) (Supplementary Figure S2). At the aa level, P1 shared high sequence identity (70.4%) with the RdRp of RLBV (Table 1).

**Table 1:** Pairwise percentage identity between amino acid sequences of putative proteins encoded by jujube yellow mottled-associated virus (JYMaV) (P1, RNA-dependent RNA polymerase; P2, glycoprotein; P3, nucleocapsid protein; P4, movement protein; P5, movement protein; P6, unknown function) and the homologous proteins encoded by emaraviruses.

Virus^1^/Protein	P1:P1	P2:P2	P3:P3	(P4/P5):P4	P6:(P5/P6/P7/P8)^2^
RLBV	70.4	52.1	65.1	63.3/24.6	3.9/24.8/26.9/38.0
HPWMoV	41.3	33.3	28.0	41.2/18.6	3.8/3.8/8.0/10.8
PVWBV	42.6	31.8	24.8	44.0/21.0	NA/NA/NA/NA
PSMoV	31.9	23.5	20.9	17.1/10.2	5.5/NA/NA/NA
RRV	32.5	21.7	20.4	18.1/10.7	5.3/7.1/5.6/NA
RYRaV	32.7	21.5	20.1	16.0/10.1	9.0/NA/NA/NA
FMV	32.8	24.1	19.3	16.5/11.4	5.0/5.9/NA/NA
BLMaV	32.2	22.1	18.9	17.3/10.6	3.3/NA/NA/NA
EMARaV	32.6	21.6	18.5	9.2/6.7	NA/NA/NA/NA
PSMoV-2	33.5	22.1	17.8	17.6/12.9	5.4/7.7/NA/NA
ACRaV	33.5	22.6	16.6	18.7/11.5	7.8/NA/NA/NA

*^*1*^EMARaV, European mountain ash ringspot-associated virus; RRV, rose rosette virus; FMV, fig mosaic virus; PPSMV and PPSMV-2, pigeonpea sterility mosaic virus and pigeonpea sterility mosaic virus 2; HPWMoV, High Plains wheat mosaic virus; RLBV, raspberry leaf blotch virus; RYRaV, redbud yellow ringspot-associated virus; AcCRaV, actinidia chlorisis ringspot-associated virus; PVWBV, Palo verde witches broom virus. ^*2*^NA, not available.*

The protein P2, translated from ORF2 (nt 2,176–191) of RNA2 (2,233 nt long), has 661 aa and an estimated molecular mass of 76.18 kDa. It shares 22.6–50.8% amino acid sequence identity with the glycoprotein precursor of emaraviruses and related viruses, and the highest similarity is with RLBV (Table 1). This protein was predicted to have five glycosylation sites (not shown) and two cleavage sites (at V_25_ES/SS and V_218_LA/DD) that would process the GP precursor into the C-terminal glycoprotein (Gc, 50.89 kDa) and two N-terminal glycoproteins, a larger Gn (22.3 kDa) and a smaller Gs (3.0 kDa). The latter cleavage site is next to a transmembrane (TM2) domain (aa 198–220) similar to other emaraviruses (Supplementary Figure S3); other two TM domains were found at aa 123–166 (TM1) and 611–630 (TM3) of the GP.

RNA3 (1,259 nt) encodes the putative protein P3 (ORF3; nt 1,189–305; 294 aa) with a molecular mass of 33.13 kDa. Sequence alignment of P3 with the NP of emaraviruses and related viruses, identified two conserved amino acid blocks (N_130_-S_133_-A_139_; N_177_-L_179_-G_198_-E_200_-P_207_-E_209_) similar to the NP motifs reported previously in other emaraviruses (Elbeaino et al., 2009b). P3 showed 65.1% amino acid sequence identity with the NP encoded by RLBV (Table 1).

**FIGURE 3:**
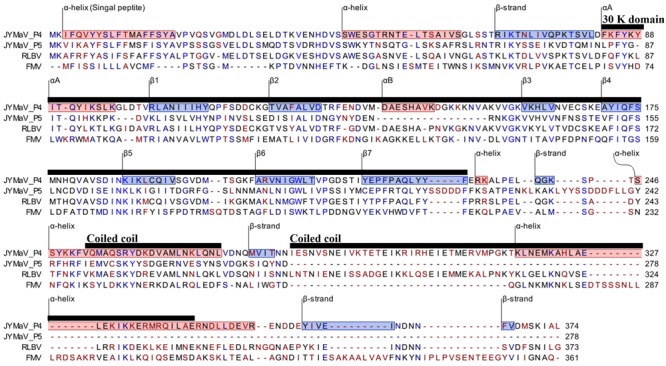
PROMALS3D alignment of P4 and P5 of jujube yellow mottled-associated virus (JYMaV) with the MPs of representative members of the genus *Emaravirus* (raspberry leaf blotch virus, RLBV, and fig mosaic virus, FMV). α-helix and β-strands are reported on red and blue background, respectively. The 30 k MP superfamily domain and the coiled coil motifs are marked by horizontal black lines.

RNA4 (1,547 nt; ORF4 from 1,446 to 322) and RNA5 (1,267 nt; ORF5 from nt 1177 to 341) encode two putative proteins (P4 and P5, respectively). P4 has 375 aa with a molecular mass of 42.73 kDa. P5 has 278 aa and a molecular mass of 31.88 kDa. These two proteins seem to be somehow related to each other: they share sequence identity of 35.2% at the N-terminus, while in the C-terminus P5 shows a large deletion, including a coiled coil region, with respect to P4. Both proteins contain a similar single signal-peptide in the N-terminus and display a secondary structure pattern composed of a series of α-helices and β-strands, namely αA, β1, β2, αB and β3–β7 from the N to the C terminus (Figure 3), which is a typical feature of 30 K movement protein (MP) superfamily (Melcher, 2000; Ishikawa et al., 2013; Yu et al., 2013). Interestingly, JYMaV P4 protein shares 63.3% aa sequence identity with the protein encoded by the RNA4 of RLBV, which has been shown to be a MP (Yu et al., 2013). JYMaV P5 protein, mainly due to the terminal deletion, shares only 24.6% sequence identity with the protein encoded by RLBV RNA4 (Table 1), Altogether, these data suggest that P4, and likely P5, could be viral MPs.

RNA6 is only 980 nt long and contains the smallest JYMaV ORF (ORF6, between positions 891 and 307). This RNA codes for the putative protein P6 (194 aa and 22.86 kDa) that has a slight sequence identity (24.4–37.8% identity) with the proteins encoded by RNAs 6–8 of RLBV (Table 1) (Lu et al., 2015). Similarly, to the latter proteins, the functional role of JYMaV P6 remains unknown.

### Phylogenetic Analysis

**FIGURE 4:**
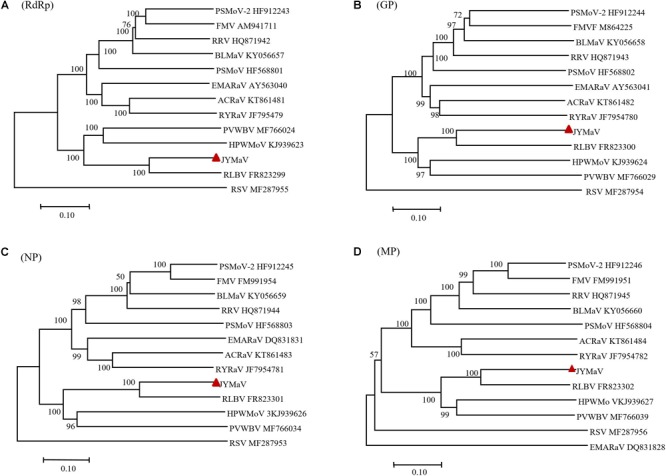
Phylogenetic trees inferred with the putative RdRP **(A)**, GP **(B)**, NP **(C)**, and MP **(D)** of jujube yellow mottled-associated virus (JYMaV), the homologous proteins of the other emaraviruses and of rice stripe virus (RSV, genus *Tenuivirus*), used as the outgroup. The accession numbers of the virus sequences used to generate each tree are reported, while JYMaV is indicated by a red triangle. The tree was generated by MEGA 7 (MUSCLE alignment, neighbor-joining and *p*-distance model, and 1000 bootstrap replicate, with values below 50% not shown).

Phylogenetic trees, inferred from amino acid sequence alignments of the P1 (RdRp), P2 (GP), P3 (NP), and P4 (MP) of JYMaV and the related proteins encoded by extant emaraviruses and other unclassified emaraviruses (BLMaV and PVWBV), showed similar topology (Figure 4). Interestingly, the clades corresponding to JYMaV, RLBV, HPWMoV, and PVWBV were always grouped together into one main branch in all phylogenetic trees, indicating a closer evolutionary link to each other, likely due to their origin from a common ancestor.

### Virus Particle Identification by TEM

**FIGURE 5:**
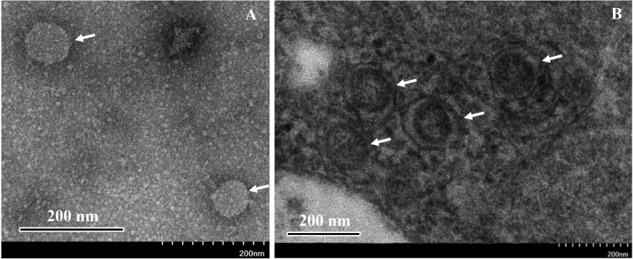
Negative staining observation of the extracts **(A)** and thin section **(B)** of fresh leaves from symptomatic jujube yellow mottled-associated virus-infected jujube tree. Virus-like particles (VLPs) and double-membrane- bound bodies (DMBs) are indicated by arrows.

Transmission electron microscopy observation of negative stained samples from symptomatic jujube trees showed spherical virus-like particles (VLPs) 80–100 nm in size (Figure 5A). As counterparts of the VLPs, the ultrathin sections of symptomatic jujube leaf tissues showed clear double-membrane-bound bodies (DMBs), approximately 100 nm in diameter, located in close proximity of the membranes of the endoplasmic reticulum of mesophyll cells (Figure 5B). Similar DMBs and cytoarchitecture profiles were observed in plant cells infected by other emaraviruses (Mielke-Ehret and Mühlbach, 2012). The VLPs, and DMBs structures were never observed in the extracts and tissues of non-symptomatic jujube, supporting their close association with the presence of both JYMaV and JYMD.

### Close Association Between JYMaV and JYMD

Besides Liaoning province, JYMD has not reported in other Chinese provinces, so far. Therefore, to perform a preliminary study on the association of the disease with JYMaV, fruit and leaf samples from 45 jujube trees grown in Liaoning province, and showing the typical symptoms of JYMD, were collected and tested by RT-PCR using JYMaV-specific primers targeting a fragment of RNA3 (Supplementary Figure S5). The virus was detected in 37 symptomatic trees (82.2%). In contrast, only one of a total of 33 symptomless jujube trees (15 trees grown in Liaoning province and 18 grown in Hebei province where the disease is not present), resulted infected by this virus when tested using the same detection method. The amplicons of the expected size (245 bp) from all the positive samples were purified and sequenced, confirming that they actually were the cDNAs fragments of the targeted JYMaV RNA3. Multiple alignments of these amplicons showed that they shared more than 98% sequence identity with each other and with the reference JYMaV genomic RNA, suggesting a relatively low sequence variability in this genomic RNA fragment among the tested virus isolates. Altogether data of this preliminary survey strongly support the association of JYMaV with JYMD.

## Discussion

Application of HTS technologies in virus diagnostics has become frequent in the last few years (Wu et al., 2015), increasing the capability of identifying previously unreported viruses as the agents of many economically important diseases in fruit trees (Maliogka et al., 2018). To investigate the etiology of JYMD, a new virus-like disease affecting jujube trees observed for the first time in 2015 in Liaoning province, we first sequenced by HTS small RNA libraries from symptomatic and symptomless jujube plants and, then, implemented these analyses with data from RNA-seq of an rRNAs-depleted total RNA library from a symptomatic plant. This strategy allowed the identification of six genomic segments of the novel negative-stranded RNA virus JYMaV in the symptomatic jujube tree.

The six genomic JYMaV RNAs have been completely sequenced using two-step RT-PCR, molecular cloning and conventional Sanger sequencing techniques, showing partial but significant sequence identity with genomic RNAs of emaraviruses, especially RLBV. Indeed, RNA 1, 2, 3, and 4 of JYMaV encode proteins that share the highest sequence identity with those encoded by the corresponding RNAs of RLBV. The other two JYMaV RNA segments (5 and 6) also recall RNAs already reported in RLBV (RNAs 4, 6, 7, or 8), although in this case the proteins encoded by the respective RNAs share only a limited sequence identity (<25%), Additional solid evidence was provided that RNA 1–6 of JYMaV are the genomic components of a single viral entity because they (i) contain the same conserved nucleotides at the respective 5′ and 3′ termini, and (ii) have been always and simultaneously detected in association with each other in at least twelve plants tested by RT-PCR using specific primers designed in the conserved termini of the JYMaV genomic RNAs.

This detection method has also the potentiality of amplifying other genomic fragments of JYMaV, besides the six ones already identified by HTS. This possibility has been taken into consideration because RLBV, the closest relative of JYMaV, has a genome composed of eight segments. However, no additional JYMaV genomic segment has been identified by the previous RT-PCR approach nor by the RNA-seq of the rRNA-depleted total RNA library from the same symptomatic tree in which JYMaV was first detected by sRNA sequencing. RNA-seq was performed because, in contrast to sRNA sequencing, it allows sequencing the RNAs independently on whether RNA silencing-dependent sRNAs are generated from them and accumulate at detectable levels in the infected cells. Interestingly, whereas contigs of the six JYMaV genomic segments were identified also in the RNA-seq library, all the efforts to find contigs representing sequences of possible additional viral genomic components were unsuccessful, thus supporting the view that the genome of JYMaV could be restricted to six segments. However, we cannot exclude that potential additional JYMaV genomic component(s) escaped detection by HTS approaches. In fact, an intrinsic limitation of HTS methods, when they are applied to detect novel viruses, is that the final result depends on whether the generated contigs share sequence identity with previously annotated viral sequences. Therefore, for JYMaV, as well as for many other emaraviruses, the possibility exists that genomic components remained unnoticed because of their high divergence from any other viral sequence available in the database.

Based on sequence analysis, JYMaV genomic RNAs are predicted to code for RdRp (P1), GP (P2), NP (P3), two putative MPs (P4 and P5), and a protein (P6) with a still unknown functional role. The question of the possible existence of two MPs in the case of JYMaV is intriguing and possibly due the modular evolution proposed for most eukaryotic viruses (Koonin et al., 2015). In the case of emaraviruses, evidence for segment reassortment, likely during mixed infections, has been recently provided for emaravirus species associated with sterility mosaic disease of pigeonpea, (Patil et al., 2017). In addition, long-playing modular transitions driven by different molecular events (recombination, reassortment, gene duplication) could explain the presence of RNA segments coding for homologous proteins in the genome of several emaraviruses, such as HPWMoV (two NPs) (Tatineni et al., 2014), RRV (RNA 5 and 7) (Di Bello et al., 2015), RLBV (RNA 6–8) (Lu et al., 2015) and JYMaV (RNA 4 and 5).

Phylogenetic trees inferred with aa sequences of JYMaV putative RdRp, NP, and those of the related proteins from other emaraviruses showed the close phylogenetic relationship between JYMaV and RLBV. In fact, these two viruses always clustered together independently on the considered protein. The species demarcation criteria in the genus *Emaravirus* are that the amino acid sequence of RdRp, GP, and NP genes between viruses belonging to distinct species must differ by 25% (Elbeaino et al., 2018). The sequence divergence of the putative RdRp, GP, and NP encoded by JYMaV with respect to all the other emaraviruses was always higher that 25% (Table 1), thus indicating that JYMaV is a member of a novel species in the genus *Emaravirus*. As for other emaraviruses, VLPs and DMBs of JYMaV were observed by TEM in extracts and in cells from symptomatic jujube leaf tissues, respectively. Membranes surrounding the DMBs were mainly found close to plant intracellular membranes (endoplasmic reticulum and Golgi cisternae) as described for tospoviruses (Kikkert et al., 1999) and other emaraviruses (Martelli et al., 1993; Kumar et al., 2002; Zheng et al., 2016).

RT-PCR amplification methods based on the primers designed in the NP gene have been previously used to detect other emaraviruses in plants (Kallinen et al., 2009). We developed a two-step RT-PCR based on a primer pair designed in the JYMaV RNA3 (coding for the NP) taking into consideration the sequence variability in the coding region of this RNA observed in the several isolates sequenced in this study. The preliminary survey showed a high JYMaV infection rate (82.2%) of symptomatic trees, thus strongly supporting the close association of the virus with JYMD. Such an association is reinforced by the almost complete absence of JYMaV in trees not affected by the disease. The single symptomless, but JYMaV-infected plant found in the Liaoning province could correspond to new infection with symptoms yet to develop or, alternatively, to a tolerant cultivar or to a plant infected by JYMaV mild isolate. Additional studies are also needed to provide the conclusive proof that JYMaV is the causative agent of JYMD, which requires fulfillment of Koch’s postulates, a complex task for negative-stranded RNA viruses, especially in the case of woody hosts that are frequently infected by more than a single graft-transmissible agent.

The availability of an efficient detection method of JYMaV will allow monitoring the geographic distribution of this virus in Liaoning province and in other Chinese provinces in which jujube trees are cultivated, thus providing additional information on the sequence and biological variability among different field isolates and on the existence of JYMaV-tolerant/resistant jujube cultivars.

## Data Availability

The datasets generated for this study can be found in the NCBI database GenBank, accession numbers from MK305894 to MK305899.

## Author Contributions

CY and MC conceived and designed the experiments. CY and JF collected the samples. CY, SZ, TH, JF, and MC conducted the experiments and analyzed data. CY, SZ, FDS, and MC discussed the results, and drafted and revised the manuscript. All authors read and approved the final draft of the manuscript.

## Conflict of Interest Statement

The authors declare that the research was conducted in the absence of any commercial or financial relationships that could be construed as a potential conflict of interest.

## Footnotes

1 https://talk.ictvonline.org/ictv-reports/ictv_online_report/

2 https://www.ncbi.nlm.nih.gov/genome/15586

3 http://www.ncbi.nlm.nih.gov/Blast.cgi

4 https://www.ncbi.nlm.nih.gov/orffinder/

5 https://www.ncbi.nlm.nih.gov/Structure/cdd/wrpsb.cgi/

6 http://annie.bii.a-star.edu.sg/annie/home.do

7 http://prodata.swmed.edu/promals3d/promals3d.php

## Supplementary Material

FIGURE S1 RT-PCR amplification and RACE strategy to generate 18 overlapping amplicons (dot lines) and obtain the whole genome of jujube yellow mottled-associated emaravirus (JYMaV). The amplification products (dotted, black lines below the genomic RNAs), obtained using forward and reverse primers designed in selected viral contigs are indicated by numbers (from 1A to 6B; the sequence of the specific primers used to amplify each cDNA are reported in Supplementary Table S1). The cDNAs 1B–E, 2B,C were amplified to confirm the presence of JYMaV in several symptomatic jujube trees. Short lines above the genomic RNAs correspond to the generated contigs, with the red and green colors discriminating the viral and complementary strand polarity of the contigs, respectively.

FIGURE S2 Schematic representation of the multiple alignment of JYMaV P1 and the RdRp of the other emaraviruses: ACRaV (KT861481), BLMaV (KY056657), EMARaV (AY563040) FMV (AM941711), HPWMoV (KJ939623), PPSMV (HF568801), PPSMV-2 (HF912243), PVWBV (MF766024), RLBV (FR823299), RRV (HQ871942), RYRaV (JF795479). The conserved core RdRp motifs and the endonuclease domain are reported and their relative positions indicated by red vertical lines.

FIGURE S3 Multiple alignment of the TM regions identified in P2 of JYMaV and in glycoproteins of emaraviruses. The TM regions (in red), the surface-exposed regions (in blue) and the clearage sites are shown based on the SignalP analysis.

FIGURE S4 RT-PCR analysis of JYMaV full-length genomic RNA components. Lane M (left): DL15,000 DNA marker (Takara, Japan), with fragment sizes reported on the left; J, JYMaV-infected jujube; H, non-infected (healthy) jujube; M (right), DL2,000 DNA marker (Takara) with fragment sizes reported on the left. The bands of expected size corresponding to genomic RNA 2–6 of JYMaV are indicated. After sequencing, the amplicon corresponding to the band of about 0.7 kb has been identified as the cDNA of a host RNA.

FIGURE S5 RT-PCR assay of JYMaV infection in jujube plants using the primer pair R3NP-R and R3NP-F. M. DNA marker; 1–20. Tested samples; 20. Positive control (JYMaV infected tree); 21. negative control (tree non infected by JYMaV); 22. Water control.

TABLE S1 Primers used in this study.

## References

[B1] Cao M. Du P. Wang X. Yu Y. Qiu Y. Li W. (2014). Virus infection triggers widespread silencing of host genes by a distinct class of endogenous siRNAs in Arabidopsis. *Proc. Natl. Acad. Sci. U.S.A.* 111 14613–14618. 10.1073/pnas.1407131111 25201959 PMC4209997

[B2] Di Bello P. L. Ho T. Tzanetakis I. E. (2015). The evolution of emaraviruses is becoming more complex: seven segments identified in the causal agent of Rose rosette disease. *Virus Res.* 210 241–244. 10.1016/j.virusres.2015.08.009 26278379

[B3] Di Bello P. L. Laney A. G. Druciarek T. Ho T. Gergerich R. C. Keller K. E. (2016). A novel emaravirus is associated with redbud yellow ringspot disease. *Virus Res.* 222 41–47. 10.1016/j.virusres.2016.05.027 27262621

[B4] Du K. Liu S. Chen Z. Fan Z. Wang H. Tian G. (2017). Full genome sequence of jujube mosaic-associated virus, a new member of the family Caulimoviridae. *Arch. Virol.* 162 3221–3224. 10.1007/s00705-017-3438-6 28612117

[B5] Elbeaino T. Digiaro M. Alabdullah A. De S. A. Minafra A. Mielke N. (2009a). A multipartite single-stranded negative-sense RNA virus is the putative agent of fig mosaic disease. *J. Gen. Virol.* 90 1281–1288. 10.1099/vir.0.008649-0 19264612

[B6] Elbeaino T. Digiaro M. Martelli G. P. (2009b). Complete nucleotide sequence of four RNA segments of fig mosaic virus. *Arch. Virol.* 154 1719–1727. 10.1007/s00705-009-0509-3 19777155

[B7] Elbeaino T. Digiaro M. Mielke-Ehret N. Mühlbach H.-P. Martelli G. P. (2018). ICTV virus taxonomy profile: fimoviridae. *J. Gen. Virol.* 99 1478–1479. 10.1099/jgv.0.001143 30204080 PMC12662054

[B8] Elbeaino T. Digiaro M. Uppala M. Sudini H. (2014). Deep sequencing of pigeonpea sterility mosaic virus discloses five RNA segments related to emaraviruses. *Virus Res.* 188 27–31. 10.1016/j.virusres.2014.03.022 24685674

[B9] Elbeaino T. Digiaro M. Uppala M. Sudini H. (2015). Deep sequencing of dsRNAs recovered from mosaic-diseased pigeonpea reveals the presence of a novel emaravirus: pigeonpea sterility mosaic virus 2. *Arch. Virol.* 160 2019–2029. 10.1007/s00705-015-2479-y 26060057

[B10] Gao Q. Wu C. Wang M. (2013). The jujube (Ziziphus jujuba Mill.) fruit: a review of current knowledge of fruit composition and health benefits. *J. Agric. Food Chem.* 61 3351–3363. 10.1021/jf4007032 23480594

[B11] Grabherr M. G. Haas B. J. Yassour M. Levin J. Z. Thompson D. A. Amit I. (2011). Trinity: reconstructing a full-length transcriptome without a genome from RNA-Seq data. *Nat. Biotechnol.* 29 644–652. 10.1186/1471-2105-12-S14-S2 21572440 PMC3571712

[B12] Gupta A. K. Hein G. L. Graybosch R. A. Tatineni S. (2018). Octapartite negative-sense RNA genome of high plains wheat mosaic virus encodes two suppressors of RNA silencing. *Virology* 518 152–162. 10.1016/j.virol.2018.02.013 29499560

[B13] Hassan M. Di Bello P. L. Keller K. E. Martin R. R. Sabanadzovic S. Tzanetakis I. E. (2017). A new, widespread emaravirus discovered in blackberry. *Virus Res.* 235 1–5. 10.1016/j.virusres.2017.04.006 28396285

[B14] Ilyas M. Avelar A. S. Schuch U. Brown J. K. (2018). First report of an emaravirus associated with witches broom disease and eriophyid mite infestations of the blue palo verde tree in Arizona. *Plant Dis.* 102:1863. 10.1094/pdis-01-18-0124-pdn 30125163

[B15] Ishikawa K. Maejima K. Komatsu K. Kitazawa Y. Hashimoto M. Takata D. (2012). Identification and characterization of two novel genomic RNA segments of fig mosaic virus, RNA5 and RNA6. *J. Gen. Virol.* 93 1612–1619. 10.1099/vir.0.042663-0 22513386

[B16] Ishikawa K. Maejima K. Komatsu K. Netsu O. Keima T. Shiraishi T. (2013). Fig mosaic emaravirus p4 protein is involved in cell-to-cell movement. *J. Gen. Virol.* 94 682–686. 10.1099/vir.0.047860-0 23152372

[B17] Juan R. Friedemann W. Stephen C. (2010). Bunyaviridae RNA polymerases (L-protein) have an N-terminal, influenza-like endonuclease domain, essential for viral cap-dependent transcription. *PLoS Pathog.* 6:e1001101. 10.1371/journal.ppat.1001101 20862319 PMC2940753

[B18] Kallinen A. Lindberg I. Tugume A. Valkonen J. (2009). Detection, distribution, and genetic variability of European mountain ash ringspot-associated virus. *Phytopathology* 99 344–352. 10.1094/PHYTO-99-4-0344 19271975

[B19] Kikkert M. Van Lent J. Storms M. Bodegom P. Kormelink R. Goldbach R. (1999). Tomato spotted wilt virus particle morphogenesis in plant cells. *J. Virol.* 73 2288–2297. 9971812 10.1128/jvi.73.3.2288-2297.1999 PMC104474

[B20] Koonin E. V. Dolja V. V. Krupovic M. (2015). Origins and evolution of viruses of eukaryotes: the ultimate modularity. *Virology* 479 2–25. 10.1016/j.virol.2015.02.039 25771806 PMC5898234

[B21] Kormelink R. Garcia M. L. Goodin M. Sasaya T. Haenni A. L. (2011). Negative-strand RNA viruses: the plant-infecting counterparts. *Virus Res.* 162 184–202. 10.1016/j.virusres.2011.09.028 21963660

[B22] Kumar P. L. Duncan G. Roberts I. Teifion Jones A. Reddy D. (2002). Cytopathology of pigeonpea sterility mosaic virus in pigeonpea and *Nicotiana benthamiana*: similarities with those of eriophyid mite-borne agents of undefined aeti- ology. *Ann. Appl. Biol.* 140 87–96. 10.1111/j.1744-7348.2002.tb00160.x

[B23] Kumar S. Stecher G. Tamura K. (2016). MEGA7: molecular evolutionary genetics analysis version 7.0 for bigger datasets. *Mol. Biol. Evol.* 33 1870–1874. 10.1093/molbev/msw054 27004904 PMC8210823

[B24] Laney A. G. Keller K. E. Martin R. R. Tzanetakis I. E. (2011). A discovery 70 years in the making: characterization of the rose rosette virus. *J. Gen. Virol.* 92 1727–1732. 10.1099/vir.0.031146-0 21471323

[B25] Liu M. Zhao J. Cai Q. Liu G. Wang J. Zhao Z. (2014). The complex jujube genome provides insights into fruit tree biology. *Nat. Commun.* 5:5315. 10.1038/ncomms6315 25350882 PMC4220462

[B26] Lu Y. McGavin W. Cock P. J. Schnettler E. Yan F. Chen J. (2015). Newly identified RNAs of raspberry leaf blotch virus encoding a related group of proteins. *J. Gen. Virol.* 96 3432–3439. 10.1099/jgv.0.000277 26358478

[B27] Maes P. Adkins S. Alkhovsky S. V. Avsic-Zupanc T. Ballinger M. J. Bente D. A. (2019). Taxonomy of the order bunyavirales: second update 2018. *Arch. Virol.* 164 927–941. 10.1007/s00705-018-04127-3 30663021 PMC6581445

[B28] Maliogka V. Minafra A. Saldarelli P. Ruiz-García A. Glasa M. Katis N. (2018). Recent advances on detection and characterization of fruit tree viruses using high-throughput sequencing technologies. *Viruses* 10:436. 10.3390/v10080436 30126105 PMC6116224

[B29] Martelli G. P. Castellano M. A. Lafortezza R. (1993). An ultrastructural study of fig mosaic. *Phytopathol. Mediterr.* 32 33–43. 10.1128/JVI.02527-14 25320328 PMC4301128

[B30] Martelli G. P. Russo M. (1984). Use of thin sectioning for visualization and identification of plant viruses. *Methods Virol.* 8 143–224. 10.1016/b978-0-12-470208-0.50011-6

[B31] McGavin W. J. Mitchell C. Cock P. J. Wright K. M. MacFarlane S. A. (2012). Raspberry leaf blotch virus, a putative new member of the genus Emaravirus, encodes a novel genomic RNA. *J. Gen. Virol.* 93 430–437. 10.1099/vir.0.037937-0 22049090

[B32] Melcher U. (2000). The ‘30K’ superfamily of viral movement proteins. *J. Gen. Virol.* 81 257–266. 10.1099/0022-1317-81-1-257 10640565

[B33] Mielke-Ehret N. Mühlbach H.-P. (2007). A novel, multipartite, negative-strand RNA virus is associated with the ringspot disease of European mountain ash (*Sorbus aucuparia* L.). *J. Gen. Virol.* 88 1337–1346. 10.1099/vir.0.82715-0 17374780

[B34] Mielke-Ehret N. Mühlbach H.-P. (2012). Emaravirus: a novel genus of multipartite, negative strand RNA plant viruses. *Viruses* 4 1515–1536. 10.3390/v4091515 23170170 PMC3499817

[B35] Ooi H. S. Kwo C. Y. Wildpaner M. Sirota F. L. Eisenhaber B. Maurer-Stroh S. (2009). ANNIE: integrated de novo protein sequence annotation. *Nucleic Acids Res.* 37 435–440. 10.1093/nar/gkp254 19389726 PMC2703921

[B36] Patil B. L. Dangwal M. Mishra R. (2017). Variability of emaravirus species associated with sterility mosaic disease of pigeonpea in India provides evidence of segment reassortment. *Viruses* 9:183. 10.3390/v9070183 28696402 PMC5537675

[B37] Patil B. L. Kumar P. L. (2015). Pigeonpea sterility mosaic virus: a legume-infecting Emaravirus from South Asia. *Mol. Plant Pathol.* 16 775–786. 10.1111/mpp.12238 25640756 PMC6638375

[B38] Pei J. Kim B. H. Grishin N. V. (2008). PROMALS3D: a tool for multiple protein sequence and structure alignments. *Nucleic Acids Res.* 36 2295–2300. 10.1093/nar/gkn072 18287115 PMC2367709

[B39] Tatineni S. McMechan A. J. Wosula E. N. Wegulo S. N. Graybosch R. A. French R. (2014). An eriophyid mite-transmitted plant virus contains eight genomic RNA segments with unusual heterogeneity in the nucleocapsid protein. *J. Virol.* 88 11834–11845. 10.1128/JVI.01901-14 25100845 PMC4178757

[B40] Wu Q. Ding S. Zhang Y. Zhu S. (2015). Identification of viruses and viroids by next-generation sequencing and homology-dependent and homology-independent algorithms. *Annu. Rev. Phytopathol.* 53 425–444. 10.1146/annurev-phyto-080614-120030 26047558

[B41] Yang I. Hafner G. Dale J. Harding R. (2003). Genomic characterisation of taro bacilliform virus. *Arch. Virol.* 148 937–949. 10.1007/s00705-002-0969-1 12721801

[B42] Yu C. Karlin D. G. Lu Y. Wright K. Chen J. MacFarlane S. (2013). Experimental and bioinformatic evidence that raspberry leaf blotch emaravirus P4 is a movement protein of the 30K superfamily. *J. Gen. Virol.* 94 2117–2128. 10.1099/vir.0.053256-0 23761405

[B43] Zerbino D. R. (2010). Using the velvet de novo assembler for short-read sequencing technologies. *Curr. Protoc. Bioinformatics* Chapter 11:Unit11.5. 10.1002/0471250953.bi1105s31 20836074 PMC2952100

[B44] Zhang S. Shen P. Li M. Tian X. Zhou C. Cao M. (2018). Discovery of a novel geminivirus associated with camellia chlorotic dwarf disease. *Arch. Virol.* 163 1709–1712. 10.1007/s00705-018-3780-3 29500570

[B45] Zheng Y. Navarro B. Wang G. Wang Y. Yang Z. Xu W. (2016). Actinidia chlorotic ringspot-associated virus: a novel emaravirus infecting kiwifruit plants. *Mol. Plant Pathol.* 18 569–581. 10.1111/mpp.12421 27125218 PMC6638214

