# Supplementary figures and images for: Identification and Characterization of a Novel Emaravirus Associated With Jujube (Ziziphus jujuba Mill.) Yellow Mottle Disease
Source: Front Microbiol. 2019 Jun 25;10:1417. doi: 10.3389/fmicb.2019.01417 (PMC6603204; doi:10.3389/fmicb.2019.01417)

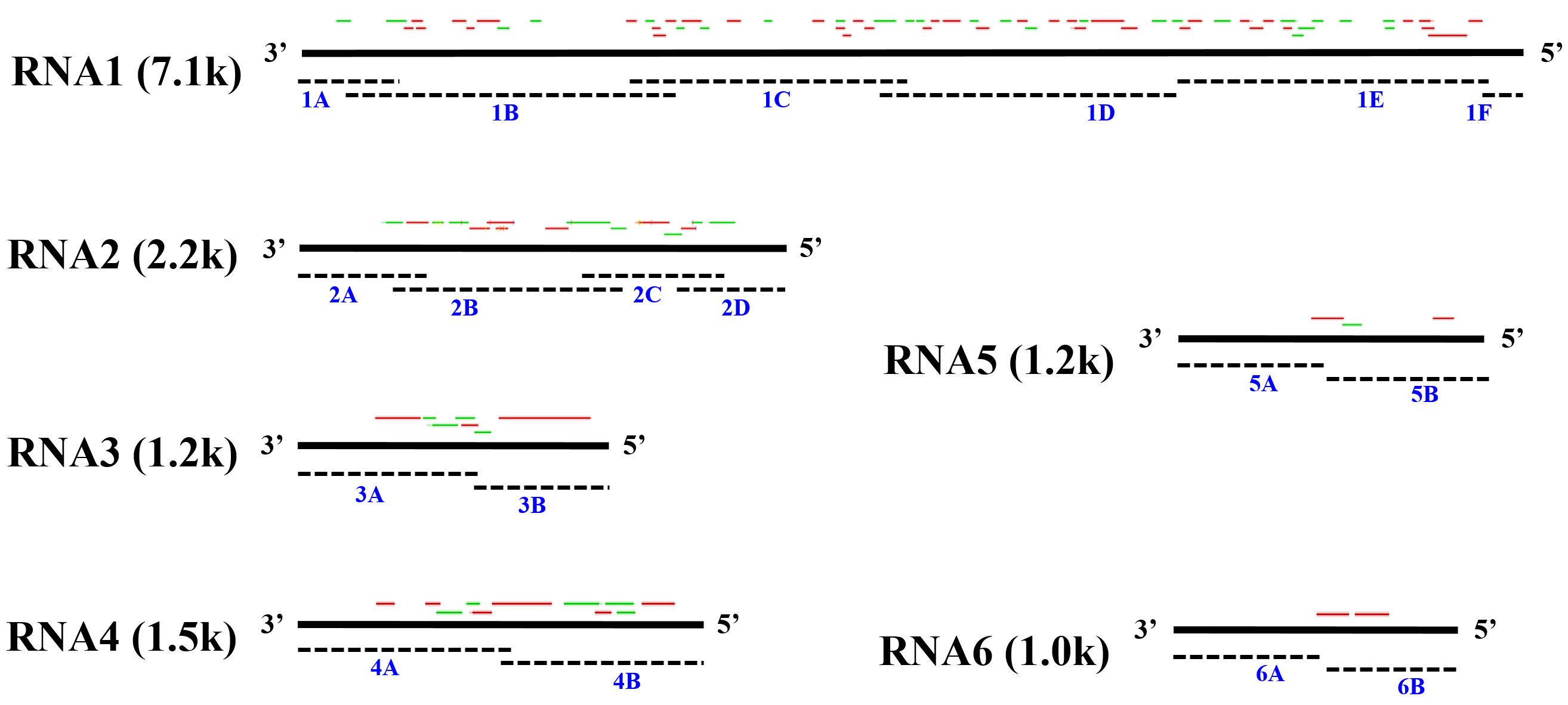

Supplement: FIGURE S1 — RT-PCR amplification and RACE strategy to generate 18 overlapping amplicons (dot lines) and obtain the whole genome of jujube yellow mottled-associated emaravirus (JYMaV). The amplification products (dotted, black lines below the genomic RNAs), obtained using forward and reverse primers designed in selected viral contigs are indicated by numbers (from 1A to 6B; the sequence of the specific primers used to amplify each cDNA are reported in Supplementary Table S1). The cDNAs 1B–E, 2B,C were amplified to confirm the presence of JYMaV in several symptomatic jujube trees. Short lines above the genomic RNAs correspond to the generated contigs, with the red and green colors discriminating the viral and complementary strand polarity of the contigs, respectively. [file Image_1.JPEG]

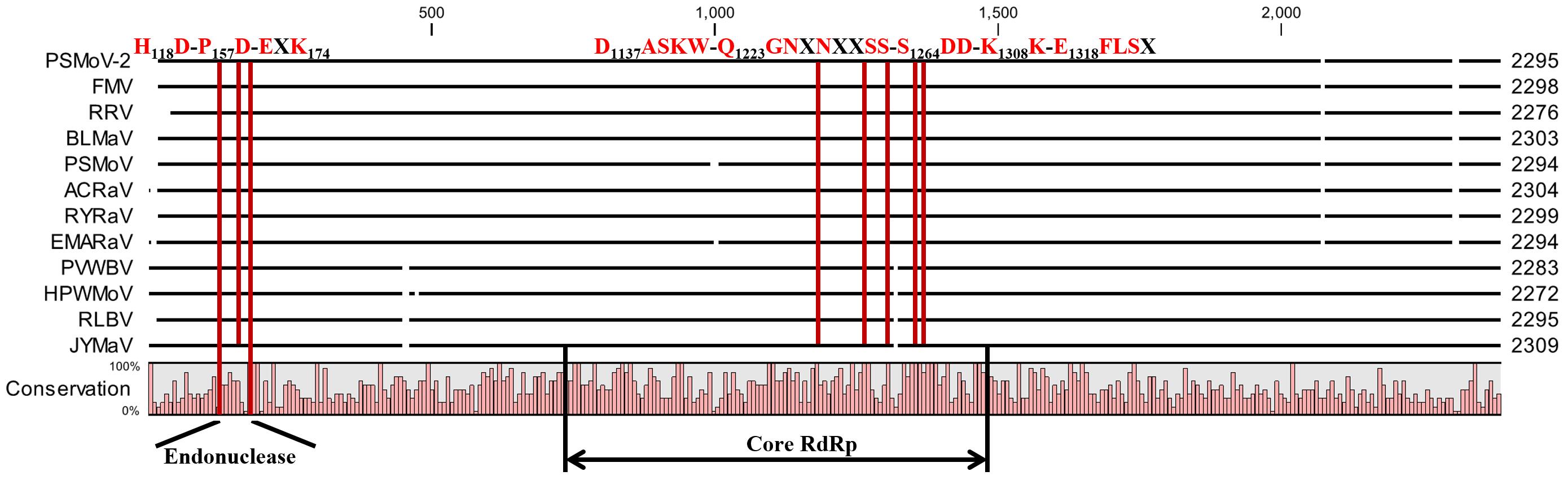

Supplement: FIGURE S2 — Schematic representation of the multiple alignment of JYMaV P1 and the RdRp of the other emaraviruses: ACRaV (KT861481), BLMaV (KY056657), EMARaV (AY563040) FMV (AM941711), HPWMoV (KJ939623), PPSMV (HF568801), PPSMV-2 (HF912243), PVWBV (MF766024), RLBV (FR823299), RRV (HQ871942), RYRaV (JF795479). The conserved core RdRp motifs and the endonuclease domain are reported and their relative positions indicated by red vertical lines. [file Image_2.JPEG]

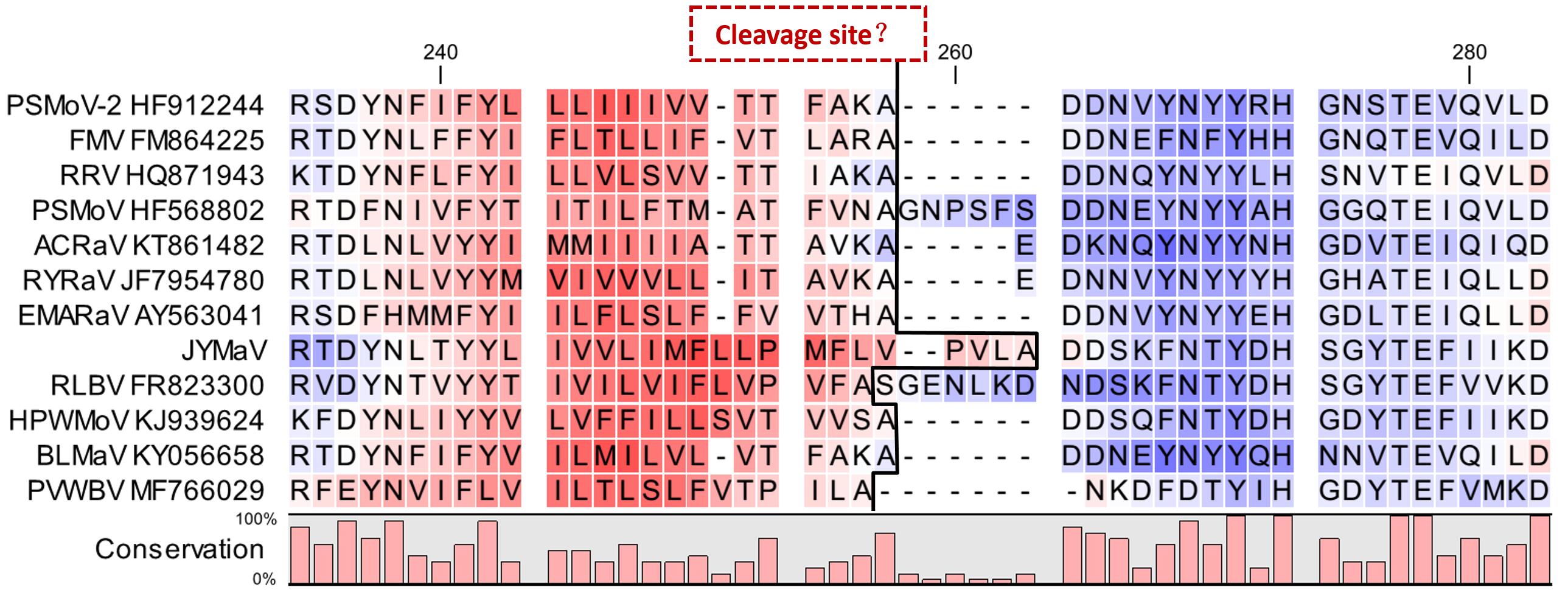

Supplement: FIGURE S3 — Multiple alignment of the TM regions identified in P2 of JYMaV and in glycoproteins of emaraviruses. The TM regions (in red), the surface-exposed regions (in blue) and the clearage sites are shown based on the SignalP analysis. [file Image_3.JPEG]

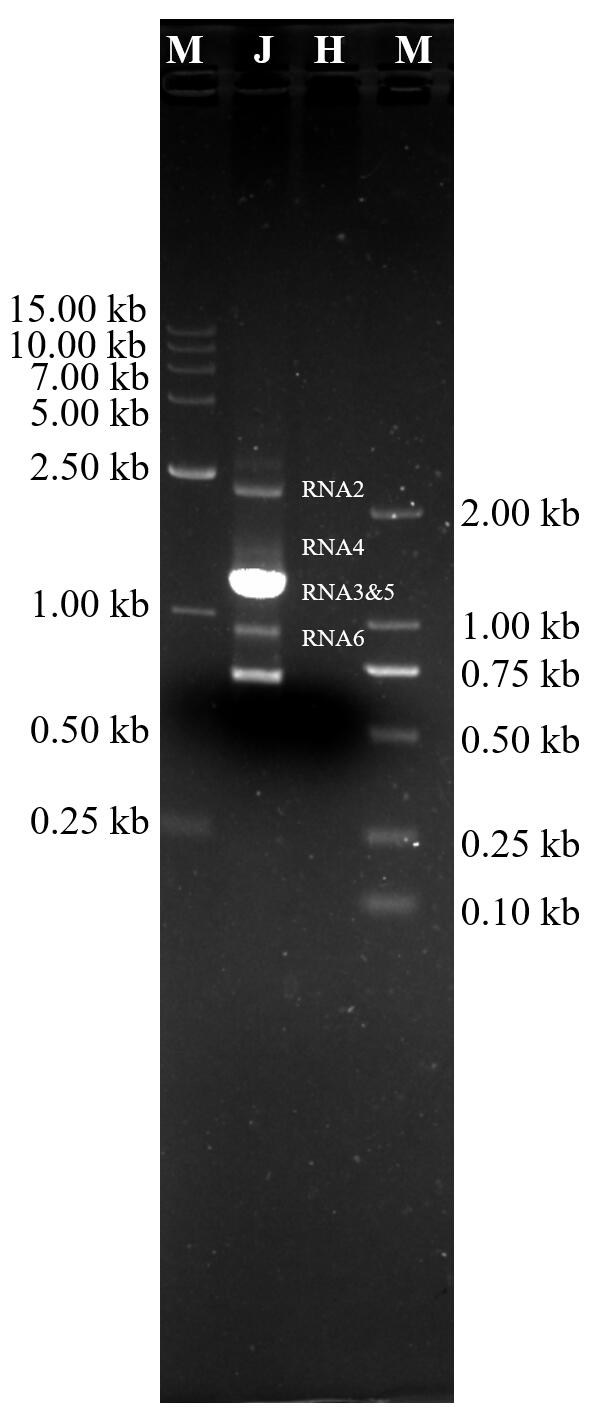

Supplement: FIGURE S4 — RT-PCR analysis of JYMaV full-length genomic RNA components. Lane M (left): DL15,000 DNA marker (Takara, Japan), with fragment sizes reported on the left; J, JYMaV-infected jujube; H, non-infected (healthy) jujube; M (right), DL2,000 DNA marker (Takara) with fragment sizes reported on the left. The bands of expected size corresponding to genomic RNA 2–6 of JYMaV are indicated. After sequencing, the amplicon corresponding to the band of about 0.7 kb has been identified as the cDNA of a host RNA. [file Image_4.JPEG]

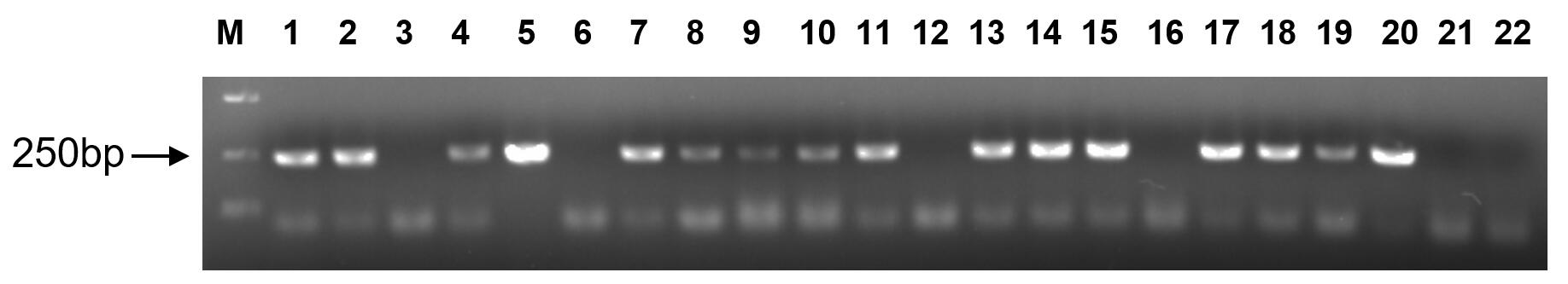

Supplement: FIGURE S5 — RT-PCR assay of JYMaV infection in jujube plants using the primer pair R3NP-R and R3NP-F. M. DNA marker; 1–20. Tested samples; 20. Positive control (JYMaV infected tree); 21. negative control (tree non infected by JYMaV); 22. Water control. [file Image_5.JPEG]
