# Supplementary material for: Identification and Characterization of a Novel Emaravirus Associated With Jujube (Ziziphus jujuba Mill.) Yellow Mottle Disease
Source: Front Microbiol. 2019 Jun 25;10:1417. doi: 10.3389/fmicb.2019.01417 (PMC6603204; doi:10.3389/fmicb.2019.01417)
Supplement: TABLE S1 — Primers used in this study. [file Table_1.DOCX]

**Supplementary table**

**Identification and characterization of a novel emaravirus associated with** **jujube** **(*****Ziziphus jujuba* Mill.) yellow mottle disease**

Caixia Yang^1 &^*, Song Zhang^2,3 &^, Tong Han^1^, Jingjing Fu^1^, Francesco Di Serio^4^*, Mengji Cao^2,3^ *

^1^ Liaoning Key Laboratory of Urban Integrated Pest Management and Ecological Security, College of Life Science and Engineering, Shenyang University, Dadong, Shenyang 110044, Liaoning, China.

^2^ National Citrus Engineering and Technology Research Center, Citrus Research Institute, Southwest University, Beibei, Chongqing 400712, China;

^3^ Academy of Agricultural Sciences, Southwest University, Beibei, Chongqing 400715, China;

^4^ Istituto per la Protezione Sostenibile delle Piante, Consiglio Nazionale delle Ricerche, Bari 70126, Italy.

^&^ Co-first author

* Corresponding authors: [caomengji@cric.cn](mailto:caomengji@cric.cn);

[xueyang27@126.com](mailto:xueyang27@126.com);

francesco.diserio@ipsp.cnr.it.

| **Targeted RNA** | **Product name** | **Primer name** | **Nucleotide sequence 5’ 3’** | **Position (nt)** | **Product size (bp)** |
| --- | --- | --- | --- | --- | --- |
| RNA1 | 1A | GSP-5R-Cont160 | CTCGAACCTGACTACATTTTTGAAC | 577 | 577 |
|  | 1B | Cont92-F | ATCAAAATCGGATATGGTGGTAC | 225 | 2,139 |
|  |  | Cont78-R | TGATGAACACAGTGAGATCGAAGA | 2,363 |  |
|  | 1C | Cont310-F | TCATTCCTACTAATCCTTTGCCA | 2,028 | 1,563 |
|  |  | Cont258-R | TGTTCCAATATCAACTTGTCCCT | 3,590 |  |
|  | 1D | Cont171-F | GCCTTGTAACCAGTTTGACCTAA | 3,371 | 1,788 |
|  |  | Cont45-R | GGCATTTATGCAGGACATCTTAG | 5,158 |  |
|  | 1E | Cont45-F | TTTTTGTTCCTAAGATGTCCTGC | 5,127 | 1,808 |
|  |  | Cont132-R | TTGGATGATCCCACAAATAAGCT | 6,934 |  |
|  | 1F | GSP-3F-Cont132 | TGAACCTAGCAGCCTCAAGAATT | 6,881 | 263 |
| RNA2 | 2A | GSP-5R-Cont140 | GCTGTGGTAGGATTGATTGTGCC | 603 | 603 |
|  | 2B | Cont107-F | GATCGTTAAGTGTCACAGTAAATTC | 454 | 1,007 |
|  |  | Cont297-R | AGATGTGGAACACGAAATAAACG | 1,460 |  |
|  | 2C | Cont48-F | ATCTGAAAGTTTTGTTGTCACCCT | 1,252 | 734 |
|  |  | Cont169-R | AGCTTGTGTAAAATCGCATAAATG | 1,985 |  |
|  | 2D | GSP-3F-Cont141 | AACCCTGAATAGATGTAAAGCACCT | 1,706 | 528 |
| RNA3 | 3A | GSP-5R-Cont242 | CATTCTGTTCCTGAAGGAGTGGGCA | 685 | 685 |
|  | 3B | GSP-3F-Cont242 | CTAATTTGTTGCCCACTCCTTCAGG | 652 | 608 |
| RNA4 | 4A | GSP-5R-Cont53 | ATTTGCTAGAGTCAACATTGGCTGG | 838 | 838 |
|  | 4B | GSP-3F-Cont53 | GATAGTTGAATCACCAGGCACCGTC | 787 | 761 |
| RNA5 | 5A | GSP-5R-Cont177 | AATAGGTATCATAACCGATGGGAGG | 671 | 671 |
|  | 5B | GSP-3F-Cont177 | GTTCAAACTTCCAAACCTCCCATCG | 632 | 636 |
| RNA6 | 6A | GSP-5R-Cont64 | CGAATCTCGAATACTGGGACAAACT | 521 | 521 |
|  | 6B | GSP-3F-Cont64 | GAAGTTTGTCCCAGTATTCGAGATT | 495 | 486 |

**TABLE S1 | Primers used in this study.**
